# Community health workers: essential in ensuring primary health care for equitable universal health coverage, but more knowledge and action is needed

**DOI:** 10.1186/s12875-023-02175-6

**Published:** 2023-10-25

**Authors:** Lisa R Hirschhorn, Indiran Govender, Joseph M Zulu

**Affiliations:** 1 Department of Medical Social Sciences, Havey Institute for Global Health, Feinberg School of Medicine, Northwestern University, Chicago, IL USA; 2 Department of Family Medicine and Primary Health Care, Sefako Makgatho Health Sciences University, Ga-Rankuwa, Gauteng Province South Africa; 3 School of Public Health, University of Zambia, Lusaka, Zambia

## Abstract

Community health workers (CHWs) have helped improve access to quality primary health care (PHC). However, knowledge gaps exist in designing and implementing CHW-engaged models needed to ensure quality people-centered PHC. In this collection, we call for papers which bridge this knowledge gap, to build sustainable, resilient and equitable CHW programs.

The importance of integrated, quality and people-centered primary healthcare is widely recognized as critical to achieve the health-related Sustainable Development Goals (SDGs) through effective, affordable, equitable and quality universal health coverage (UHC). Community health workers (CHWs) are critical to the work needed to achieve this goal in many countries, particularly in low and middle income countries (LMICS) [1]. Growing evidence has found that CHWs have been able to advance population well-being through extending promotive, preventive and curative health services, and increasingly responding to growing challenges including the rise of non-communicable disease [2]. CHWs workers also play vital roles in responding to health system shocks such as the Ebola epidemic and COVID-19 pandemic including maintaining essential health during pandemics, conducting surveillance, increasing community trust in care, and serving as a critical link between health care facilities and the community [3].

Research has shown the need to ensure that these CHWs are salaried, supervised, supplied and trained to deliver the care needed for their community [1, 4]. Organizations and countries are moving forward in expanding this cohort needed to participate in the delivery of health care contributing to the integrated people-centered quality primary care. Key roles where CHWs have found to be effective include active population outreach to identify individuals in need of care, strengthen retention in care for the continuity needed for people with chronic illnesses including those lost in follow-up [5]. CHWs are also needed to address the large gaps in human resources for health in primary care, estimated to be 10 million by 2030 [6]. CHWs can also focus on populations that are not well served by the current PHC systems, including the elderly and adolescents.

There has also been growing work in integrating innovations in how CHWs are trained, supported and how they can deliver care and how the quality of care delivered is measured [7, 8]. Examples include digital health technology to provide ongoing training, facilitate data collection for program improvement and supervision [9]. Other opportunities include expanding innovations into communities distant from health centers such as better BP monitors, oxygen saturation measurements and point of care diagnostics. However, there remains significant gaps in the knowledge of how to best train, support and sustain CHWs. Questions include what is the optimal scope of work and how can their scope be expanded to meet emerging and currently unmet health needs, and where can innovations be adapted and strategies developed to implement these important tools available in health centers into communities [2]. Other unanswered questions include how to optimally measure and ensure the quality of care delivered, as well as insights and evidence on strategies needed to motivate, supervise and monitor CHWs and their career pathways. Implementation research is also needed to understand what are the optimal models and implementation strategies based on different contexts in which they work such as rural versus urban, and in fragile and conflict affected areas. This use of implementation research can help to identify and understand what works and importantly inform scale. Finally, we need to know what are effective funding approaches, and how to more strongly incorporate this critical cadre in the work to increase health system resilience and preparedness.

In this collection focusing on CHWs, we call for papers on the broad spectrum of the work and role of CHWs in different geographic settings and the potential of CHW in improving access to quality universal. Priorities are ones which answer these and other questions around the models and strategies needed for a sustainable, equitable and robust CHW program as a part of integrated people-centered PHC which reflect the context in which they work. The need for this knowledge has never been more urgent as the scope of PHC continues to expand to meet the needs for chronic disease management, mobile communities, and growing threats from climate change and current and new pandemics.

## Data Availability

Not applicable.
